# Changes of liver enzymes and bilirubin during ischemic stroke: mechanisms and possible significance

**DOI:** 10.1186/1471-2377-14-122

**Published:** 2014-06-06

**Authors:** Antonio Muscari, Andrea Collini, Elisa Fabbri, Marco Giovagnoli, Chiara Napoli, Valentina Rossi, Luca Vizioli, Andrea Bonfiglioli, Donatella Magalotti, Giovanni M Puddu, Marco Zoli

**Affiliations:** 1 Stroke Unit – Department of Internal Medicine, Aging and Nephrological Diseases, S. Orsola-Malpighi Hospital, Via Albertoni, 15, Bologna 40138, Italy; 2 Department of Medical and Surgical Sciences, University of Bologna, Bologna, Italy

**Keywords:** Bilirubin, C-reactive protein, γGT, GOT, GPT, Ischemic stroke, Liver

## Abstract

**Background:**

Small changes of bilirubin and liver enzymes are often detected during the acute phase of stroke, but their origin and significance are still poorly understood.

**Methods:**

On days 0, 3, 7, and 14 after admission, 180 patients with ischemic stroke underwent serial determinations of bilirubin, GOT, GPT, γGT, alkaline phosphatase, C-reactive protein (CRP) and complete blood count. On days 0 and 7 common bile duct diameter was measured by ultrasound, and on day 3 cerebral infarct volume (IV) was calculated from CT scan slices.

**Results:**

During the first week GOT, GPT, γGT (P < 0.001) and CRP (P = 0.03) increased with subsequent plateau, while significant decrements (P < 0.001) concerned unconjugated bilirubin, erythrocytes and haemoglobin. Alkaline phosphatase, direct bilirubin and common bile duct diameter remained stable. IV correlated with CRP, leukocytes, GOT, γGT (r > 0.3, P < 0.001 for all) and direct bilirubin (r = 0.23, P = 0.008). In multivariate analysis only CRP and GOT remained independently associated with IV (P < =0.001). The correlation of IV with GOT increased progressively from admission to day 14. GOT independently correlated with GPT which, in turn, correlated with γGT. γGT was also highly correlated with leukocytes. Unconjugated bilirubin correlated with haemoglobin, which was inversely correlated with CRP.

**Conclusions:**

The changes of bilirubin and liver enzymes during ischemic stroke reflect two phenomena, which are both related to IV: 1) inflammation, with consequent increment of CRP, leukocytes and γGT, and decrease of haemoglobin and unconjugated bilirubin and 2) an unknown signal, independent from inflammation, leading to increasing GOT and GPT levels.

## Background

Some previous studies have reported an increment of bilirubin levels during ischemic or haemorrhagic stroke, and their association with the severity of symptoms [1,2]. These results (concerning in particular direct bilirubin [1]), could be related to the anti-oxidant action of this molecule, which might counteract the oxidative processes contributing to cerebral damage during the acute phase [3,4].

Changes during the acute phase of stroke have also been reported for gamma-glutamyl-transferase (γ-GT) [5], an enzyme that is typically associated with cholestasis and alcohol consumption. In particular, γ-GT might increase due to a contraction of the sphincter of Oddi caused by alterations of the autonomic nervous system, or by a fast-related reduction of cholecystokinin secretion [5]. But even a direct release of γ-GT and alkaline phosphatase (ALP) from cerebral lesions cannot be ruled out, as these enzymes are present in the endothelium of cerebral capillaries and therefore they might behave as markers of blood–brain barrier lesion [6]. In addition, γ-GT would have a long term predictive significance for new cases of stroke [7,8], which could be due to its relation to alcohol, which in turn is a recognized risk factor for stroke [9,10].

In the Atorvastatin During Ischemic Stroke (ADIS) study, a double-blind controlled randomized investigation, we assessed the short-medium term effects of the administration of high-dose atorvastatin during the first week of ischemic stroke [11]. In the two groups of patients very significant (although not very wide) increments of glutamate-oxaloacetate-transaminase (GOT), glutamate-piruvate-transaminase (GPT) and γ-GT serum levels were detected during the week of study. The increment of transaminases, in particular, which had previously been reported only sporadically and mainly in the cerebrospinal fluid [12,13], occurred in most patients of both groups.

Overall, these data suggest that, beyond the explanations so far proposed to justify the increases in bilirubin and γ-GT, there may be a true involvement of the liver in stroke. The present prospective study was performed to describe more precisely the changes of bilirubin and liver enzymes in the acute phase of ischemic stroke, and to search for their possible explanations, considering in particular the relationships with cerebral lesion volume, markers of inflammation and common bile duct diameter.

## Methods

### Patients

The study started on April 1, 2011, and the last patient was enrolled on June 27, 2012. During that period the patients with suspected stroke (sudden and persistent focal neurological deficit) were admitted to the Emergency Department of our Hospital, where they underwent the first laboratory assessment and CT scan, and were possibly treated with rT-PA. After a variable period of time (depending on the type of treatment and the time of admission, see Table 1) 313 patients with suspected ischemic stroke were transferred to our Stroke Unit. Subsequently, 133 of them were excluded due to the following criteria: thrombolytic treatment (possible interference with serum turnover of enzymes and bilirubin, 29% of exclusions), diagnosis of TIA or other disease different from ischemic stroke (25%), delay from onset of symptoms > 72 hours (17%), refusal to provide the informed consent (11%), HBsAg or anti-HCV positivity (10%), alcohol abuse (>3 alcoholic units/day, 6%), or other exclusion criteria (jaundice of any type, present or previous inflammatory or neoplastic pathology of the pancreas or biliary tree, 2%). Eventually the study participants were 180 (mean age 73.2 ± 13.2 years, 53% women, see Table 1). In the ADIS Study [11], in 31 patients with ischemic stroke a mean increment of 5 ± 8 U of both GOT and γ-GT was obtained after 7 days from admission (P = 0.001). Hypothesizing, for the present study, a mean increment of only 3 ± 8 U with a P value of 0.01, 110 patients would be needed to get a power of 90% (a size well below the number of our patients, even considering missing data).

**Table 1:** General characteristics of the study population (N = 180)

**Characteristic**	**Value**
Age (years)	73.2 ± 13.2
Male sex	85 (47.2)
Cerebral lesion volume (ml)	4.7 [0.4-35.5]
Admission to emergency dept. delay (hrs)*	1.7 [0.8-6.3]
Admission to stroke unit delay (hrs)*	16.8 [6–28.7]
OCSP classification	
LACS	32 (17.8)
PACS	65 (36.1)
TACS	55 (30.6)
POCS	28 (15.6)
TOAST classification	
Large artery	28 (15.6)
Cardioembolism	57 (31.5)
Small artery	45 (25.0)
Other cause	6 (3.3)
Undetermined	44 (24.4)
Initial NIHSS score	6 [3-15]
<10	112 (62.2)
> = 10 and < 18	36 (20.0)
> = 18	32 (17.8)
Diabetes	41 (22.8)
Hypertension	152 (84.4)
Current smoker	35 (19.4)
Ex-smoker	31 (17.2)
Hypercholesterolemia	112 (62.2)
Malignant neoplasm	12 (6.7)
Previous myocardial infarction	32 (17.8)
Previous stroke	29 (16.1)
Previous TIA	9 (5.0)
Statin (pre-admission)	46 (25.6)
Clopidogrel (pre-admission)	14 (7.8)
Oestro-progestinic (pre-admission)	2 (1.1)

Values are number (percentage), or mean ± SD, or median [25th-75th percentile].

*Delays are referred to stroke onset.

LACS = lacunar anterior circulation syndrome, NIHSS = National Institutes of Health Stroke Scale, OCSP = Oxfordshire Community Stroke Project, PACS = partial anterior circulation syndrome, POCS = posterior circulation syndrome, TACS = total anterior circulation symdrome, TOAST = Trial of ORG 10172 in Acute Stroke Treatment.

The severity of neurological deficit was assessed by the National Institutes of Health Stroke Scale (NIHSS) on admission, at discharge, and every time it was deemed necessary according to clinical picture variations.

All patients (or a relative in case of severe motor deficit or inability of understanding) signed the informed consent form. The study was approved by the joint University-Hospital Ethics Committee, and was conducted according to the criteria of the Helsinki Declaration.

### Study variables

On admission to Stroke Unit (median delay from stroke onset 16.8 hours, see Table 1), and after 7 days, the patients underwent a venous blood drawing for a complete blood count and the determination of the following serum parameters: GOT, GPT, ALP, γ-GT, total biliribin, direct bilirubin (unconjugated bilirubin = total – direct blirubin) and C-reactive protein (CRP). In addition, at the same times 2 abdominal ultrasound assessments were performed, for the measurement of common bile duct and inferior vena cava diameter, and the search for possible intrahepatic bile duct dilatation, liver steatosis and gallbladder stones.

Moreover, on days 3 and 14 the measurements of liver markers (GOT, GPT, ALP, γ-GT and fractionated bilirubin) were repeated. Finally, the transaminases (GOT and GPT) were also measured on admission to the Emergency Department (median delay from stroke onset 1.7 hours, see Table 1).

All laboratory determinations were performed in the Central Laboratory of our Hospital, using automated procedures and commercially available kits: AST and ALT Cobas (UV test) for the measurement of transaminases, ALP2 Cobas (colorimetric test) for the measurement of ALP, GGT-2 Cobas (enzymatic colorimetric test) for the measurement of γ-GT, TBILI and BILD2 Cobas (method with diazoreagent) for the measurement of fractionated bilirubin, and Tina-quant CRP-Latex for the measurement of high sensitivity CRP. All kits were produced by Roche Diagnostics, Mannhein, Germany. Finally, the complete blood counts were performed by an automated counter (Bayer ADVIA 120).

Generally on admission to Stroke Unit, or on the following day, a supra-aortic ultrasound assessment was performed, while on day 3 a second brain CT scan (in addition to the one performed in the Emergency Department) was performed to precisely define the site and volume of cerebral infarct.

For each patient the infarct volume, expressed in ml, was obtained by one of the authors (A.C.) from the 3rd day scan measuring the lesion area in each CT slice, then adding up all the lesion areas, and multiplying the result by the mean slice thickness.

### Statistical analysis

The study variables are described by mean ± SD in case of normal distribution, or median and interquartile interval in case of non-gaussian distribution. The differences between means were tested by Student’s t, while the differences between medians were assessed by Mann-Whitney’s *U* test. The comparisons between 2 different times of the same variable were assessed with Student’s t for paired data or with Wilcoxon’s test, as appropriate. The differences between percentages were tested by *χ*^2^. All simple correlations were assessed by Pearson’s r coefficients after logarithmic transformation of the variables with non-gaussian distribution. Multivariate analysis was performed by multiple linear regressions and standardized β coefficients, with backward elimination of the non-significant associations. Also in this case the log-normal variables were previously log-transformed. P values < 0.05 were considered significant and two-tail tests were used throughout. The analyses were performed using SYSTAT 10 (SPSS Inc, Chicago, IL, USA).

## Results

The general characteristics of the sample population are illustrated in Table 1. Overall there was a prevalence of hypertensive, hypercholesterolemic and with mild symptoms patients. The median value of cerebral infarct volume was 4.7 ml (range 0–462.3).

### Variables changing during the acute phase of stroke

Table 2 reports the mean or median values of the study variables, both on admission and on the 7th day. Missing values increased with time, and concerned different variables from one patient to another. Thus, the variables had different sample sizes, considering also that for each variable both values (on admission and on the 7th day) had to be available.

**Table 2:** Time course of liver enzymes, bilirubin, blood counts and C-reactive protein

**Variable**	**Normal range**	**N**	**Admission**	**7th day**	**P**
GOT (U/l)	<38	138	19 [14-24]	22 [16–30]	<0.001
GPT (U/l)	<41	140	15 [11-21]	20 [13–33.5]	<0.001
γGT (U/l)	<61	138	22 [15–32]	26 [19–42]	<0.001
ALP (U/l)	<130	133	76 [59–108]	75 [62–106]	0.48
Unconjugated bilirubin (mg/dl)	<0.80	119	0.43 [0.31-0.64]	0.33 [0.23-0.43]	<0.001
Direct bilirubin (mg/dl)	<0.30	119	0.26 [0.19-0.37]	0.25 [0.18-0.33]	0.13
Erythrocytes (n/10^6^/mmc)	4.50-6.10	141	4.89 ± 0.67	4.72 ± 0.65	<0.001
Haemoglobin (g/dl)	13.0-16.5	142	13.6 ± 1.9	13.1 ± 2.2	<0.001
WBC (n/10^3^/mmc)	4.20-9.00	136	8.71 [6.95-10.41]	7.76 [6.42-9.88]	0.002
Platelets (n/10^3^/mmc)	150-380	138	224.8 ± 77.8	240.7 ± 90.6	<0.001
CRP (mg/dl)	<0.80	118	0.80 [0.27-3.58]	1.67 [0.36-4.38]	0.03

Values for admission and 7th day are medians [25th-75th percentile] or means ± SD.

ALP = Alkaline phosphatase, CRP = C-reactive protein, GOT = Glutamate oxaloacetate transaminase, GPT = glutamate piruvate transaminase, γGT = gamma glutamyl transferase, WBC = white blood cells.

Both on admission and on the 7th day the upper limit of the interquartile interval of direct bilirubin, leukocytes and CRP exceeded their maximum normal value: thus, more than a quarter of the patients had high values of these variables already on admission. GOT, GPT, γGT and platelets displayed small (generally within the normal range) but highly significant increments, while CRP had a less significant increment. On the other hand, unconjugated bilirubin, erythrocytes, haemoglobin and leukocytes decreased significantly.

The mean or median values on the 3rd and 14th day are not reported since none of them differed significantly from the mean or median values obtained, respectively, on admission and on the 7th day. Thus, during the second week all variables displayed a “plateau” course. Moreover, due to the random presence of missing values, the addition of these data to the Table would have caused a further reduction in sample size.

Since ALP and direct bilirubin did not change, they will not be further considered in the following analysis. Table 3 shows the simple correlations between the logarithm of the infarct volume and the values on admission, the values on the 7th day, and the differences between 7th day and admission, of the 9 variables that varied during the acute phase of stroke. GOT, γGT, leukocytes and CRP were directly correlated with the volume of the ischemic lesion, particularly on the 7th day. Thus, the 7th day values of the 9 variables listed in Table 3 were included in a multivariate model (multiple linear regression), with the logarithm of cerebral infarct volume as dependent variable (Table 4, N = 110). The model was also adjusted for age, sex and initial NIHSS score. After backward elimination of the non-significant associations, only log(CRP) and log (GOT) remained independently associated with the logarithm of infarct volume (overall R^2^ = 0.28, P < 0.001). Seventy patients were not included in this analysis as they were lacking of some of the above 9 variables. Thus, we compared the general characteristics (see Table 1) of these 70 patients with those of the 110 patents that had all the data. There were no significant differences for most of the variables considered, including, in particular, age, infarct volume and NIHSS score. The two groups differed only for sex (57.1% males among the patients with missing data, vs. 40.9% among the other patients, P = 0.03), and for the prevalence of previous TIA (respectively, 0 vs. 8.2%, P = 0.01).

**Table 3:** Simple correlations of the study variables with the logarithm of cerebral infarct volume

**Variable**	**Admission**			**7th day**			**Δ (7th day - Admission)**		
	**N**	**r**	**P**	**N**	**r**	**P**	**N**	**r**	**P**
Log (GOT)	172	0.24	0.002	137	0.35	<0.001	136	0.07	0.42
Log (GPT)	173	0.14	0.08	138	0.16	0.07	138	-0.00	0.96
Log (γGT)	173	0.11	0.15	136	0.34	<0.001	136	0.29	<0.001
Log (Unconjugated bilirubin)	160	0.04	0.63	129	0.04	0.63	117	-0.07	0.43
Erythrocytes	171	-0.03	0.48	141	-0.02	0.82	139	-0.03	0.68
Haemoglobin	172	-0.04	0.59	141	-0.01	0.94	140	0.04	0.64
Log (WBC)	170	0.38	<0.001	136	0.36	<0.001	134	0.08	0.37
Platelets	172	0.08	0.28	137	0.07	0.42	136	0.02	0.83
Log (CRP)	163	0.33	<0.001	123	0.41	<0.001	116	0.29	0.002

Only the variables with significant changes during the first week are shown.

CRP = C-reactive protein, GOT = Glutamate oxaloacetate transaminase, GPT = glutamate piruvate transaminase, γGT = gamma glutamyl transferase, WBC = white blood cells.

**Table 4:** Significant multivariate associations between the study variables (7th day determination) and the logarithm of cerebral infarct volume

**Variable**	**β**	**S.E.**	**Standardized β**	**P**
Log (GOT)	1.367	0.557	0.200	0.01
Log (CRP)	0.565	0.171	0.310	0.001
Age	-0.011	0.022	-0.048	0.61
Male sex	-0.520	0.500	-0.085	0.30
Log (Initial NIHSS score)	0.518	0.188	0.240	0.007
Intercept	-3.090	2.466	-	0.213

Result of a multiple linear regression (N = 110) with the logarithm of cerebral infarct volume as dependent variable and all variables listed in Table 3 as initial independent variables, after backward elimination of non-significant associations. The model includes also age, sex and initial NIHSS score as adjustment factors.

CRP = C-reactive protein, GOT = Glutamate oxaloacetate transaminase, NIHSS = National Institutes of Health Stroke Scale.

**Figure 1:**
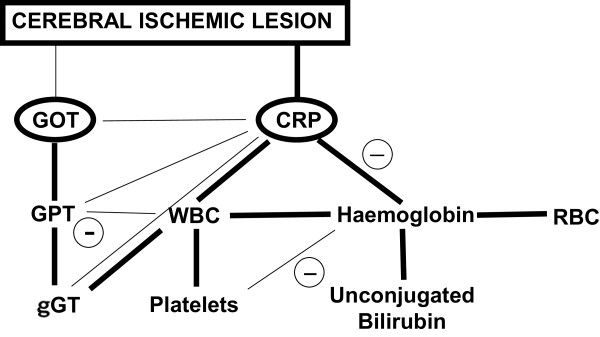
**Schematic representation of the independent associations with infarct volume reported in Table **4**and of the independent associations among the study variables reported in Table **5**.** The strongest associations (standardized β > =0.3, P < 0.001) are shown in bold. Minus signs indicate inverse associations. CRP = C-reactive protein, GOT = Glutamate oxaloacetate transaminase, GPT = glutamate piruvate transaminase, γGT = gamma glutamyl transferase, RBC = red blood cells, WBC = white blood cells.

The 9 variables that changed during the acute phase were variously correlated to each other. Table 5 shows, and Figure 1 graphically outlines, the independent correlations of each of these variables with the other variables (multiple linear regressions with backward elimination of non-significant associations). The figure also reports the independent associations with cerebral infarct volume. All the variations of the variables considered in this study seem to be attributable to 2 main changes, namely those concerning GOT and CRP, which in turn were the only variables independently associated with cerebral infarct size. Inflammation is responsible for the increase in CRP, leukocytes and platelets, and also contributes to the increase in γGT. In addition, CRP is inversely associated with hemoglobin, which progressively decreases together with red blood cells and unconjugated bilirubin (see also Table 2).

**Table 5:** Significant multivariate associations among the study variables (7th day determination, N = 112)

**Dependent variable**	**Independent variables**									
	**Log (GOT)**	**Log (GPT)**	**Log (γGT)**	**Log (Unc.Bil.)**	**Erythrocytes**	**Hb**	**Log (WBC)**	**Platelets**	**Log (CRP)**	**R**^**2**^
Log (GOT)		0.78^c^							0.18^b^	0.62^c^
Log (GPT)	0.65^c^		0.34^c^						-0.27^c^	0.69^c^
Log (γGT)		0.54^c^					0.33^c^		0.19^a^	0.51^c^
Log (Unc.Bil.)						0.52^c^				0.27^c^
Erythrocytes						0.82^c^				0.68^c^
Haemoglobin				0.19^b^	0.74^c^					0.71^c^
Log (WBC))		-0.19^a^	0.34^c^			0.31^c^		0.30^c^	0.52^c^	0.61^c^
Platelets						-0.23^a^	0.38^c^			0.18^c^
Log (CRP)						-0.34^c^	0.65^c^			0.50^c^

Results of multiple linear regressions (standardized β coefficients), with backward elimination of non-significant associations.

^a^P < 0.05, ^b^P < 0.005, ^c^P < 0.001.

CRP = C-reactive protein, GOT = Glutamate oxaloacetate transaminase, GPT = glutamate piruvate transaminase, γGT = gamma glutamyl transferase, WBC = white blood cells.

GOT levels are independently influenced by cerebral infarct volume, but are also associated with CRP levels (β = 0.18). It is therefore possible that the effect of a variable on GOT levels may vary in relation to the variations of the other variable. To test this hypothesis we constructed a linear regression model including log(GOT) as dependent variable, and log (infarct volume), log(CRP) and log(infarct volume) X log(CRP) (interaction term) as independent variables. In this analysis only log(infarct volume) remained associated with log(GOT) (β = 0.26, P = 0.007), while both log(CRP) (β = -0.011, P = 0.92) and the interaction term (β = 0.112, P = 0.25) were not significant, so that the hypothesis of an interaction between the 2 variables (infarct volume and CRP) on GOT levels was rejected.

Thus, the association between GOT and infarct volume seems to be independent from inflammation, and appears of particular interest. The correlation between the logarithm of infarct volume and the logarithm of GOT was not present on admission to the Emergency Department (median delay from onset of symptoms 1.7 hours; r = 0.13; P = 0.11; N = 158), it was first detectable on admission to Stroke Unit (median delay 16.8 hours; r = 0.24; P = 0.002; N = 172), it increased on the 7th day (r = 0.35; P < 0.001; N = 137), and was maximum on the 14th day (r = 0.50; P < 0.001; N = 95).

### Direct bilirubin

As above reported, direct bilirubin did not change significantly between admission and 7th day. However, on the 7th day a significant correlation (N = 129, r = 0.23, P = 0.008) was observed between the logarithm of direct bilirubin and the logarithm of infarct volume, which was not present on admission. But when the logarithm of direct bilirubin was included in the multivariate analysis of the factors associated with the logarithm of infarct volume, this variable was immediately eliminated, showing that its association with infarct volume was not independent.

The multivariate search for the factors associated with direct bilirubin showed its independent associations with the logarithm of unconjugated bilirubin (P < 0.001), the logarithm of γGT (P = 0.001) and the logarithm of common bile duct diameter (P = 0.03).

### Ultrasound parameters

None of the ultrasound variables considered in this study (common bile duct diameter, inferior vena cava diameter and intrahepatic bile duct dilatation) changed significantly between admission and 7th day. With the exception of the above reported correlation between the logarithm of common bile duct diameter and the logarithm of direct bilirubin, no correlations were found among ultrasound parameters and the laboratory variables assessed in this study. Among the patients with available measurements of common bile duct diameter, plus γ-GT and GOT on the 7th day (N = 94), the high quintile of common bile duct diameter (0.6-1.0 cm) consisted of 17 patients, who included 8 of the 9 patients previously subjected to cholecystectomy. The cholecystectomized patients were then excluded, and 9 patients remained with an apparently unexplained common bile duct dilatation. The Glasgow Coma Score of these patients was significantly lower than that of the rest of the sample: 10 [9-15] vs. 15 [13-15], P = 0.04, but the 2 groups did not differ in the median values of γ-GT and GOT.

## Discussion

This study has shown that the small but significant changes of liver enzymes and bilirubin occurring during the early phase of ischemic stroke are attributable, directly or indirectly, to the size of cerebral infarct. To a large extent such changes are due to inflammation, but this is not the case for serum GOT, which is influenced by infarct size with mechanisms that are presently unknown.

### Inflammation, unconjugated bilirubin and γGT

The strong independent correlation of ischemic lesion volume with CRP demonstrates that inflammation during stroke course is directly proportional to cerebral damage, as already reported by Ormstad et al. [14].

Inflammation is a recognized important cause of anemia [15], deriving from at least three different mechanisms mediated by inflammatory cytokines: inhibited production of erythropoietin, reduced response of the erythroid progenitors to erythropoietin, and reduced iron release from stores caused by the polypeptide hormone hepcidin [16,17]. Also in this study, as in others previously [18], a strong inverse correlation was shown between CRP and haemoglobin. Thus, in the acute phase of stroke, in addition to the increase in CRP, white blood cells and platelets, a decrease in haemoglobin and red blood cells occurs (the small decrement of white blood cells during the first week probably follows a rapid initial increment, as suggested by the high median value of white blood cells on admission to Stroke Unit). Unconjugated bilirubin is a heme by-product, so it is directly proportional to haemoglobin. Therefore, the significant fall of unconjugated bilirubin in acute stroke seems to be a simple epiphenomenon of haemoglobin fall.

Finally, we found a strong independent association between white blood cells and γGT, so that also the increase of this enzyme in acute stroke is probably influenced by inflammation, as suggested by a few data reporting a connection between inflammation and γGT [19,20]. In previous studies γGT was found to be predictive of cardiovascular risk [21,22] and, in particular, of the risk of stroke [8] even independently of alcohol consumption [23]. Thus, the association between γGT and cardiovascular risk may be due, to a large extent, to the fact that inflammation is an important cardiovascular risk factor [24].

γGT is also a marker of cholestasis, and previously Sevastos et al. detected a common bile duct dilatation in a minority (4.1%) of stroke patients [5]. All such patients were in a deep coma, so that the authors hypothesized an inordinate hypertonia of the sphincter of Oddi induced by a severe autonomic and hypothalamic impairment. In fact our analysis confirmed that the stroke patients with a dilatation of the common bile duct that was not due to previous cholecystectomy presented a significantly more severe degree of coma than the rest of the sample. However, this aspect concerned only few patients, and could not explain the progressive increment of liver enzymes in the whole sample. In particular, no change in common bile duct diameter was found during the first 7 days in Stroke Unit, and other markers of cholestasis, such as direct bilirubin and ALP, did not show any variation.

### GOT and liver involvement

In this study GOT was the only enzyme directly and independently correlated with cerebral infarct size. This fact is in sharp contrast with the results obtained by Campos et al. [25], who found instead an *inverse* relationship between serum levels of GOT and infarct volume in patients with ischemic stroke. These authors proposed the hypothesis that GOT may exert a protective role, as it is capable of metabolizing, and therefore neutralizing, the toxic glutamate [26,27] that is released from the ischemic cerebral tissue into the blood. Thus, infarct size would be influenced by the pre-existing levels of GOT, and would be greater in the patients with a poor production of protective GOT. Our data demonstrate instead that there is a progressive increment in GOT levels, with a maximum about on the 7th day after admission and subsequent plateau, and that the correlation with infarct volume is not only direct, but also increasingly stronger with the passing of time after the acute event. Thus, our results show that GOT production is influenced by cerebral infarct volume, and not vice versa. It seems probable that some substance released from cerebral infarct and different from inflammatory cytokines (perhaps glutamate itself), is capable of stimulating the production of GOT (whose possible protective effect is however not excluded).

The differences with the study by Campos et al. [25] may have several explanations. Firstly, these authors measured GOT levels only on admission, i.e. on average in their study nearly 3 hours after stroke onset, while in the present study the strongest relationships between GOT and infarct volume were found on the 7th and 14th day. However, in our patients even when GOT was measured on admission to the Stroke Unit a significant direct relationship with infarct volume was found, while when GOT was measured in the Emergency Department (median delay from onset of symptoms 1.6 hours) no correlation with infarct volume was found, and in any case the relationship was not of inverse type. Furthermore, the sample by Campos et al. was rather different from ours, as 35% of their patients had been thrombolysed (while thrombolysed patients had been excluded from our sample), cardioembolic strokes were over-represented (50%), and lacunar strokes were under-represented (4%). Finally, in our study the infarct volume was measured in a more precise way, and log-transformed values were used in the calculation of linear regressions between non-gaussian variables.

Although GOT increments during stroke have been reported also in cerebrospinal fluid [12,13], the chained correlations among this enzyme, GPT and γGT suggest that its synthesis probably occurs in the liver. Also a direct release of GOT from necrotic cerebral tissue does not seem probable, as the appearance of the enzyme in blood does not follow a log-normal course (with early peak and subsequent progressive decrease), as is the case, for example, after a myocardial infarction. Instead the enzyme gradually accumulates in blood, with a progressive increase, and a plateau persisting at least until the 14th day.

Further studies are needed to clarify the mechanisms underlying the apparently independent association between cerebral lesion size and serum GOT levels. At present, the hypothesis that the release of toxic glutamate by the ischemic cerebral tissue [26,27] may induce the hepatic synthesis of the main enzymes involved in the metabolism of this neurotransmitter remains the most probable one.

### Direct bilirubin

Direct bilirubin displayed no variations during the acute phase of stroke. However, in some cases its levels exceeded the maximum normal value on both admission and 7th day. Furthermore, the values measured on the 7th day correlated with infarct volume, and previously direct bilirubin was found to be associated with stroke severity as assessed by the NIH stroke scale [1,28]. In our study multivariate analysis showed that the main determinants of direct bilirubin were unconjugated bilirubin and γGT: the former is evidently the substrate from which direct bilirubin originates, while the latter, during the course of stroke, is probably synthesized by the liver in response to factors including inflammation (see above), which in part might also trigger the glucuronoconjugation of bilirubin. Since during the first week after stroke onset unconjugated bilirubin decreases and γGT increases, the net result on the trend of direct bilirubin could be a plateau. Furthermore, the association of direct bilirubin with γGT may explain the association with infarct volume, and consequently with stroke severity.

### Limitations

The patients were admitted to our Stroke Unit after having spent a variable period of time in the Emergency Department. It is possible that during this early phase some markers (especially CRP, leukocytes and direct bilirubin) may have had a sharp rise that was not detected.

The measurement of serum glutamate might have allowed a better understanding of the pathophysiologic mechanisms involved.

## Conclusions

This study suggests that the liver participates to a variable extent in the acute phase of ischemic stroke, producing enzymes in response to signals coming from cerebral infarct and proportional to its size. An important signal is likely represented by inflammatory cytokines, which induce the production of acute phase reactants such as CRP and probably γGT. Another signal, presently unknown and perhaps coinciding with toxic glutamate, triggers the synthesis of GOT and indirectly of GPT. Instead, unconjugated bilirubin progressively decreases, together with haemoglobin, in response to inflammation, while direct bilirubin does not show any significant variation. Also, the hypothesis that the changes of liver enzymes and bilirubin are related to extrahepatic cholestasis was not confirmed: this phenomenon probably concerns only a small minority of comatose patients.

In conclusion, GOT is the only liver enzyme directly associated with the ischemic cerebral lesion independently from inflammation. Possibly this enzyme, neutralizing the toxic glutamate, might play a protective role, as some reports on its favourable prognostic significance suggest [25,29].

## Competing interests

The authors declare that they have no competing interest.

## Authors’ contributions

AM designed the study, analysed the data and wrote the manuscript. AC performed infarct volume measurements and participated in data analysis. EF, MG, CN, VR and LV recruited patients and significantly contributed to data collection. AB and DM performed the ultrasound investigations and critically revised the manuscript. GMP was involved in patients’ care and data analysis. MZ critically revised the manuscript. All authors read and approved the final version.

## Pre-publication history

The pre-publication history for this paper can be accessed here:

http://www.biomedcentral.com/1471-2377/14/122/prepub
